# Afamelanotide for Treatment of the Protoporphyrias: Impact on Quality of Life and Laboratory Parameters in a US Cohort

**DOI:** 10.3390/life14060689

**Published:** 2024-05-28

**Authors:** Rebecca K. Leaf, Hetanshi Naik, Paul Y. Jiang, Sarina B. Elmariah, Pamela Hodges, Jennifer Mead, John Trinidad, Behnam Saberi, Benny Tran, Sarah Valiante, Francesca Mernick, David E. Leaf, Karl E. Anderson, Amy K. Dickey

**Affiliations:** 1 Division of Hematology/Oncology, Massachusetts General Hospital, Boston, MA 02114, USA; phodges@mgb.org (P.H.); jmead1@mgb.org (J.M.); 2 Harvard Medical School, Boston, MA 02115, USA; jtrinidad@mgh.harvard.edu (J.T.); bsaberi@bidmc.harvard.edu (B.S.); deleaf@bwh.harvard.edu (D.E.L.); 3 Department of Genetics, Stanford University School of Medicine, Stanford, CA 94305, USA; naikh@stanford.edu; 4 Division of Pulmonary & Critical Care Medicine, Massachusetts General Hospital, Boston, MA 02114, USA; pyjiang@mgh.harvard.edu (P.Y.J.); btran0@mgh.harvard.edu (B.T.); 5 Department of Dermatology, Massachusetts General Hospital, Boston, MA 02114, USA; sarina.elmariah@ucsf.edu; 6 Department of Dermatology, University California San Francisco, San Francisco, CA 94143, USA; 7 Division of Gastroenterology & Hepatology, Beth Israel Deaconess Medical Center, Boston, MA 02215, USA; 8 Department of Pharmacy, Massachusetts General Hospital, Boston, MA 02114, USA; sarahvaliante@gmail.com (S.V.); fmernick@mgb.org (F.M.); 9 Division of Renal Medicine, Brigham and Women’s Hospital, Boston, MA 02115, USA; 10 Department of Internal Medicine, Division of Gastroenterology & Hepatology, University of Texas Medical Branch, Galveston, TX 77555, USA; kanderso@utmb.edu

**Keywords:** erythropoietic protoporphyria, X-linked protoporphyria, EPP, XLP, protoporphyria, cutaneous porphyria, porphyria, afamelanotide, quality of life (QoL), heme biosynthesis

## Abstract

Background: Erythropoietic protoporphyria (EPP) and X-linked protoporphyria (XLP) are rare disorders of heme biosynthesis characterized by severe cutaneous phototoxicity. Afamelanotide, an α-melanocyte-stimulating hormone analogue, is the only approved treatment for protoporphyria and leads to increased light tolerance and improved quality of life (QoL). However, published experience with afamelanotide in the US is limited. Methods: Here, we report on all adults who received at least one dose of afamelanotide at the Massachusetts General Hospital Porphyria Center from 2021 to 2022. Changes in the time to phototoxic symptom onset, QoL, and laboratory parameters were assessed before and during treatment with afamelanotide. Results: A total of 29 patients with protoporphyria were included, 26 of whom (72.2%) received ≥2 afamelanotide implants. Among the patients who received ≥2 implants, the median time to symptom onset following sunlight exposure was 12.5 min (IQR, 5–20) prior to the initiation of afamelanotide and 120 min (IQR, 60–240) after treatment (*p* < 0.001). Improvements in QoL during afamelanotide treatment were measured using two QoL tools, with good correlation observed between these two instruments. Finally, we found no improvements in the median levels of metal-free erythrocyte protoporphyrin, plasma protoporphyrin, or liver biochemistries during versus prior to the initiation of afamelanotide treatment. Conclusions: This study highlights a dramatic clinical benefit of afamelanotide in relation to light tolerance and QoL in protoporphyria, albeit without improvement in protoporphyrin levels or measures of liver function.

## 1. Introduction

Erythropoietic protoporphyria (EPP) and X-linked protoporphyria (XLP), collectively known as the protoporphyrias, are inherited photodermatoses characterized by severe cutaneous phototoxic reactions, and rarely, hepatic failure [1,2]. Management of the protoporphyrias has historically been limited to sunlight avoidance and the use of light-protective clothing; however, these measures impair quality of life (QoL). Afamelanotide, an α-melanocyte-stimulating hormone (α-MSH) analogue that increases the production of eumelanin, was approved by the US Food and Drug Administration in 2019 for patients with protoporphyria based on two phase-three clinical trials conducted in the US and the European Union [3]. However, published experience with afamelanotide in the US is limited.

## 2. Methods

Here, we report on all adults who received at least one dose of afamelanotide at the Massachusetts General Hospital (MGH) Porphyria Center over a 20-month period. This study was approved by the Mass General Brigham (MGB) Institutional Review Board. Afamelanotide (16 mg) was administered subcutaneously as frequently as every 8 weeks, with a maximum of 6 implants per year.

The outcomes included changes in time until phototoxic symptoms, QoL, and laboratory parameters before versus during afamelanotide treatment. Patients were included if they received at least one afamelanotide implant at the MGH Porphyria Center. The outcome measurements were only available for patients who returned for at least one additional afamelanotide implant after their initial dose (e.g., those who received ≥2 implants).

The metal-free erythrocyte protoporphyrin and plasma protoporphyrin levels were analyzed by Mayo Clinic Laboratories (Rochester, MN, USA) using high-performance liquid chromatography (HPLC) with fluorometry.

Patients completed two validated QoL questionnaires, the Patient-Reported Outcomes Measurement Information System 57 (PROMIS-57 v2.1) [4] and the proprietary EPP-QoL tool [5], at baseline and prior to each implant. Further, prior to initiation of afamelanotide and then at each subsequent visit, patients were asked how long it took them to develop phototoxic symptoms following sunlight exposure. Time to phototoxic symptoms was defined as the total amount of time in one day that patients could be outside in bright sunlight before cutaneous protoporphyria symptoms appeared. The analyses were conducted using RStudio software version 3.5.3, with a two-sided *p* < 0.05 considered significant. Changes in laboratory values, total QoL scores, and individual questions were evaluated using the Wilcoxon signed-rank test, with continuity correction where applicable. Correlations were evaluated using Pearson’s correlation coefficient (R) for distributions meeting the normality assumption and using Spearman’s rank correlation coefficient (Rho) otherwise. The normality assumption was assessed using Shapiro–Wilk tests and Q–Q plots.

## 3. Results

A total of 36 patients with EPP and XLP were evaluated during the period of interest, 29 of whom (*n* = 28 [EPP], *n* = 1 [XLP]) received at least one afamelanotide implant (Table 1).

**Figure 1:**
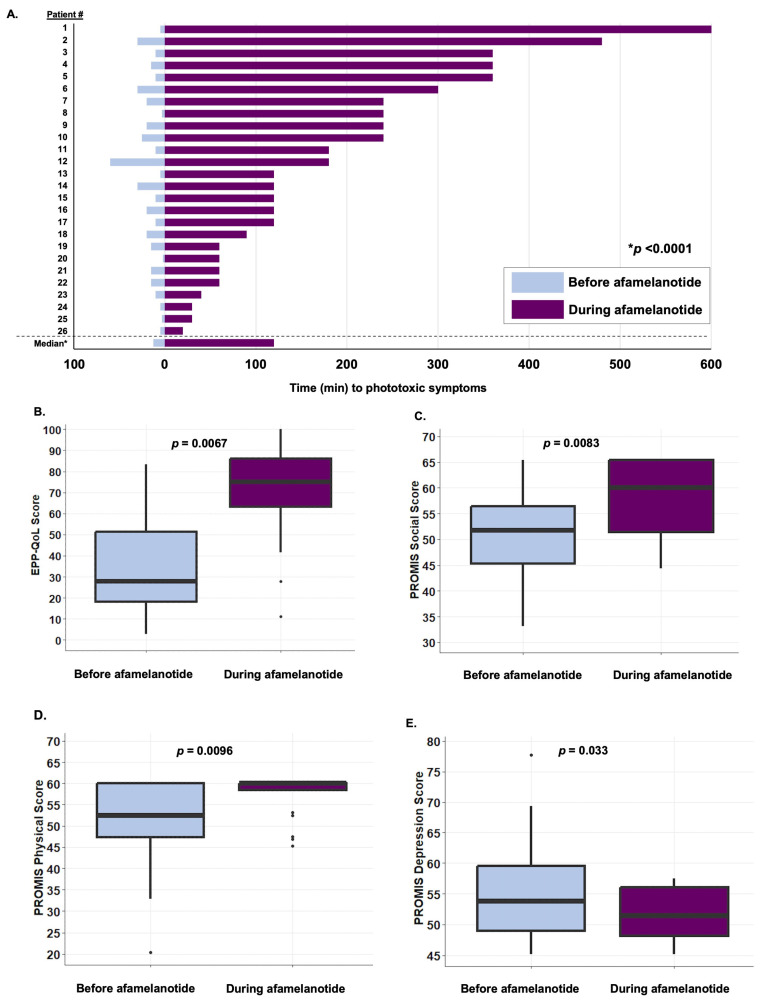
Afamelanotide is associated with an improvement in the time to phototoxic symptom onset and QoL in protoporphyria. (**A**) Time to phototoxic symptom onset (minutes) after exposure to sunlight improved during treatment with afamelanotide. (**B**) Total EPP-QoL score (ranging from 0 to 100) improved during treatment with afamelanotide. (**C**) PROMIS-57 Social Function improved during treatment with afamelanotide. (**D**) PROMIS-57 Physical Function improved during treatment with afamelanotide. (**E**) PROMIS-57 Depression decreased during treatment with afamelanotide. Data are shown as the median (IQR). Abbreviations: EPP-QoL, erythropoietic protoporphyria quality of life tool; IQR, interquartile range (25th–75th percentile); PROMIS-57, Patient-Reported Outcomes Measurement Information System 57; QoL, quality of life.

The median number of implants received over the study period was six (IQR, 2–8). Among the 26 patients who received at least 2 implants, the median time to symptom onset following sunlight exposure was 12.5 min (IQR, 5–20) prior to initiation of afamelanotide and 120 min (IQR, 60–240) after treatment (*p* < 0.0001, Figure 1A). Moreover, 100% of patients who received ≥2 doses of afamelanotide reported a decrease in the frequency and severity of their phototoxic reactions. Two patients chose not to continue treatment, both due to transportation difficulties, one of whom also reported nausea as a contributing factor.

**Figure 2:**
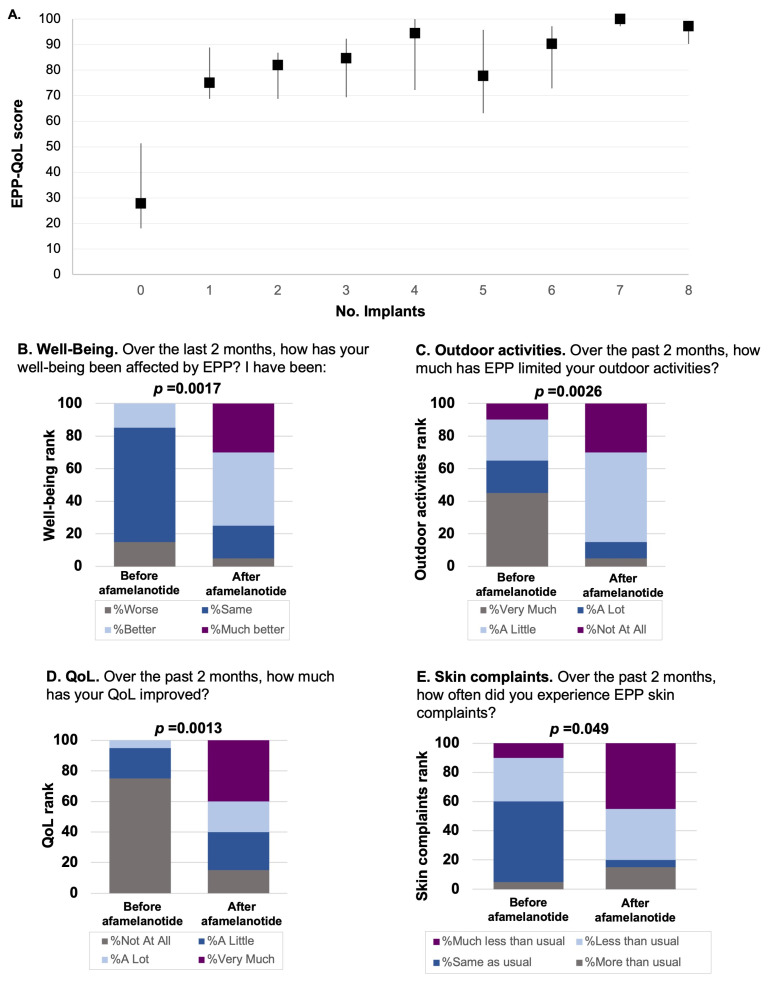
Afamelanotide is associated with improvements in the total EPP-QoL over time and within individual EPP-QoL questions. (**A**) Change in the EPP-QoL score according to the number of afamelanotide implants received. (**B**) Representative EPP-QoL question regarding well-being before and during treatment with afamelanotide. (**C**) Representative EPP-QoL question regarding outdoor activity limitations before and during treatment with afamelanotide. (**D**) Representative EPP-QoL question regarding QoL before and during treatment with afamelanotide. (**E**) Representative EPP-QoL question regarding typical EPP skin complaints before and during treatment with afamelanotide. Data are shown as the median (IQR). Abbreviations: EPP-QoL, erythropoietic protoporphyria quality of life tool; IQR, interquartile range (25th–75th percentile). QoL, quality of life.

Significant improvements in patients’ QoL were observed following the initiation of afamelanotide according to both the EPP-QoL and PROMIS-57 questionnaires. The median EPP-QoL score prior to afamelanotide initiation was 27.8 (IQR, 18.1–51.4), improving to 75 (IQR, 63.2–86.1) during treatment (*p* = 0.00067, Figure 1B), with thesechanges sustained over time (Figure 2A). Significant changes to individual questions within the EPP-QoL survey were observed as well (Figure 2B–E), with these changes again sustained over subsequent implants (Figure S1A–D).

Multiple domains of the PROMIS-57 improved during treatment with afamelanotide, including the PROMIS-57 Social Function score, the Physical Function score, and the Depression score (Figure 1C–E), with these improvements sustained over time (Figure S1E–G). Notably, the PROMIS-57 Global Pain score, which asks patients to rank their average pain over the past 7 days, worsened during treatment with afamelanotide (*p* = 0.0069, Figure S1H).

**Figure 3:**
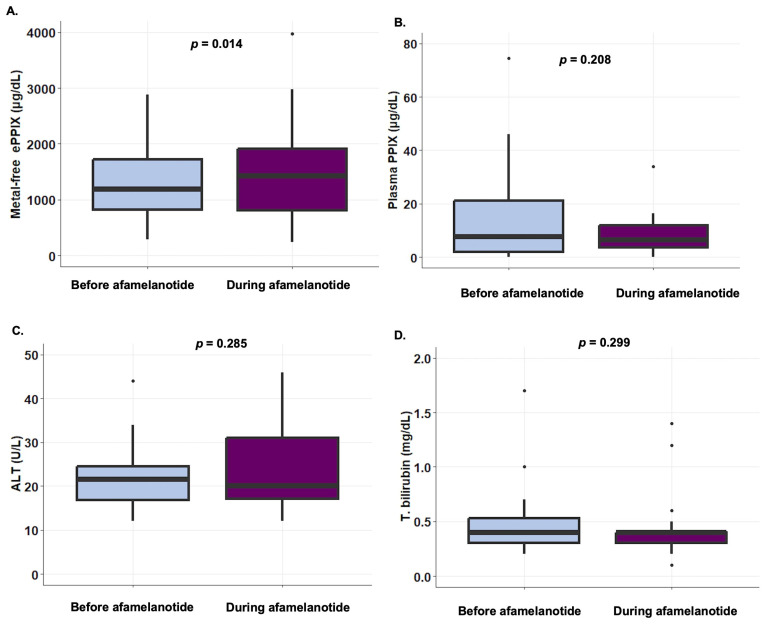
Changes in pain and biochemical studies with afamelanotide treatment. (**A**) Metal-free erythrocyte protoporphyrin levels increased slightly during afamelanotide treatment. (**B**) Plasma protoporphyrin levels did not change with afamelanotide treatment. (**C**,**D**) ALT and total bilirubin levels remained unchanged with afamelanotide treatment. Data for all the panels are shown as the median (IQR). Abbreviations: ALT, alanine transaminase; EPP-QoL, erythropoietic protoporphyria quality of life tool; ePPIX, erythrocyte protoporphyrin IX; IQR, interquartile range (25th–75th percentile); PPIX, protoporphyrin IX. QoL, quality of life.

We next assessed changes in biochemical parameters, including metal-free erythrocyte protoporphyrin, plasma protoporphyrin, aspartate aminotransferase (AST), alanine aminotransferase (ALT), and total bilirubin levels, before and during treatment with afamelanotide in 20 patients who received ≥2 implants and who had laboratory values available within 3 months of an afamelanotide dose. The median metal-free erythrocyte protoporphyrin levels increased from 1185 μg/dL (IQR, 815–1722) before treatment to 1419 μg/dL (IQR, 801–1908) during treatment (*p* = 0.014, Figure 3A). No change in median plasma protoporphyrin levels or liver biochemistries was observed before versus during treatment with afamelanotide (Figure 3B–D and Figure S3).

The change in total EPP-QoL score before and during treatment with afamelanotide correlated with corresponding changes in the PROMIS-57 Social Function score, Physical Function score, and Depression score (Figure S2A–C). The EPP-QoL survey question that asks, “Over the last two months, how much has EPP limited your amount of outdoor activities?” correlated most highly with the PROMIS-57 Social Function score (Figure S2D). Metal-free erythrocyte protoporphyrin levels were not correlated with time to symptom onset before or during afamelanotide treatment (Figure S2E,F). The time to symptom onset during afamelanotide treatment correlated moderately with the total EPP-QoL score and the PROMIS-57 Social Function and Depression domains (Figure S3G–J).

## 4. Discussion

In this cohort of 29 adults with protoporphyria in the US, afamelanotide treatment was associated with an almost 10-fold increase in the median time to phototoxic symptoms, a decrease in symptom frequency and severity, and improvements in QoL across several domains. No improvement in protoporphyrin levels or liver chemistries occurred during treatment. Afamelanotide was well-tolerated, with few side effects aside from nausea (which is well described [6,7]) and a high continuation rate.

We observed that afamelanotide was associated with an improvement in the EPP-QoL score as well as in numerous PROMIS-57 domains. When assessing individual questions of interest on the EPP-QoL survey, improvements in overall QoL, outdoor activity limitations, and the impact of EPP on well-being were all highly significant. Of the individual PROMIS-57 domain scores, the Social Function score correlated most highly with the EPP-QoL score. Additionally, we evaluated associations between time to symptom onset (a measure of light sensitivity) and QoL to understand which QoL tools best capture severity of disease. During afamelanotide therapy, time to symptoms was highly correlated with the total EPP-QoL score, the PROMIS-57 Social Function score, and the PROMIS-57 Depression score.

To more precisely assess QoL changes in patients with protoporphyria who were treated with afamelanotide, we administered both the EPP-QoL tool and the more widely available PROMIS-57 survey, which has been validated across numerous disease types. The EPP-QoL is a proprietary questionnaire originally created for clinical trial purposes and is only available by express permission [8] Therefore, this study provided an important opportunity to compare the EPP-QoL and PROMIS-57 in this patient population for the benefit of future studies in protoporphyria.

Notably, the PROMIS-57 score assessing the impact of pain and the severity of pain did not improve with afamelanotide. This effect is likely two-fold: many patients begin afamelanotide during times of the year when they are at highest risk of phototoxic reactions (e.g., spring and summertime), and also because patients may develop more symptoms in the context of testing their light-exposure limits while on a new treatment. For this reason, a global pain assessment with a short recall period by itself is not as useful for the evaluation of treatment response in patients with protoporphyria. 

Our data are consistent with other reports demonstrating a negative impact of protoporphyria on QoL [9,10] and an improvement in QoL with afamelanotide therapy [6,7]. However, real-world data on afamelanotide in the US are lacking. Two studies evaluating the post-marketing experience with afamelanotide in the US demonstrated a positive effect on patients’ well-being, but the sample sizes were small, biochemical data and light sensitivity were not reported, and no comparisons were made between QoL scores [11,12]. US-specific studies are crucial because the phase-three clinical trial of afamelanotide for the protoporphyrias demonstrated different results for US and European patients [3]. This raises questions as to whether European patients are somehow distinct from US patients due to certain environmental factors, such as weather, altitude, and latitude/longitude.

In the present study, we did not detect any decreases in protoporphyrin levels or reductions in liver biochemistry values during treatment with afamelanotide, in contrast to one European cohort, although our study population was smaller and included fewer longitudinal values [13]. In fact, we found that metal-free erythrocyte protoporphyrin levels rose slightly during treatment compared with baseline levels, which we suspect to be related to natural variation and not a medication effect. Notably, in the phase-three clinical trial that led to afamelanotide’s approval, no change in erythrocyte protoporphyrin levels was observed despite measurement with each dose [3]. Furthermore, we found no associations between metal-free erythrocyte protoporphyrin and time to symptoms or QoL, either before or during treatment with afamelanotide, which may be due to our cohort being underpowered to observe such an association. 

The limitations of this study include the modest sample size, comparison of only two time points for many of the outcomes, recall bias regarding time to symptom onset, and follow-up of just 20 months. Furthermore, we only present data on AST, ALT, and total bilirubin, and these liver biochemistries may be within the normal range even in patients with protoporphyria-related liver disease [14]. We collected these labs for clinical reasons at least once yearly, but not with each afamelanotide dose, as performed in another study [13]. Moreover, metal-free protoporphyrin levels vary by as much as 25% within the same patient over time, which could influence the variability in these results, especially as our data are presented in aggregate [15]. Changes in protoporphyrin levels may be related to iron status, liver disease, or other unknown factors, and we were unable to identify factors that would affect protoporphyrin values in our patients or control for these factors in the analysis. We also do not show protoporphyrin variation over time in the same patient in the absence of afamelanotide therapy. Additional long-term studies in a larger patient population at more sites are needed to understand the effect of afamelanotide on protoporphyrin levels and liver biochemistries, including alternative measures of hepatic function such as elastography.

In conclusion, our study demonstrates that afamelanotide increases the time to phototoxic symptom onset and QoL in patients with protoporphyria. However, we did not find significant improvements in protoporphyrin levels or liver biochemistries before versus during treatment with afamelanotide. Furthermore, because of correlations with improvements in time to symptom onset, our study confirms that both the EPP-QoL and the PROMIS-57 Social Function and Depression domains have the greatest utility in evaluating QoL and response to treatment in this patient population. Additional investigations into the effect of afamelanotide on the pathobiology of protoporphyria are needed; in particular, to better understand variability in treatment response.

## Figures and Tables

**Table 1:** Baseline characteristics of patients who received ≥1 afamelanotide implant.

Baseline Characteristics	N = 29
Age (yr), median (IQR)	40 (23–57)
Female sex, no. (%)	22 (76%)
Erythrocyte PPIX (mcg/dL), median (IQR)	1185 (815.0–1722.0)
Plasma PPIX (mcg/dL), median (IQR)	7.5 (1.9–21.1)
Aspartate aminotransferase (U/L), median (IQR)	20.50 (18.8–23.0)
Alanine aminotransferase (U/L), median (IQR)	21.5 (16.8–24.5)
Total bilirubin (mg/dL), median (IQR)	0.400 (0.30–0.53)
Time to symptom onset (min), median (IQR)	12.5 (5.0–20.0)
Number of implants, median (IQR)	6 (2–8)

Abbreviations: PPIX, protoporphyrin IX.

## Data Availability

The datasets presented in this article are not readily available due to privacy restrictions. Requests to access the datasets should be directed to the corresponding authors.

## Supplementary Materials

The following supporting information can be downloaded at: https://www.mdpi.com/article/10.3390/life14060689/s1, Figure S1: Changes in individual EPP-QoL and PROMIS-57 questions were sustained over time during treatment with afamelanotide. (A) Improvement in well-being was sustained over time. (B) Improvement in outdoor activities limitation was sustained over time. (C) Improvement in QoL was sustained over time. (D) there was a non-significant trend toward improvement in EPP skin complaints over time. (E) Improvement in Social Function was sustained over time. (F) Improvement in Physical Function was sustained over time. (G) Decrease in depression was sustained over time. (H) Pain increased over time. Data for all panels are shown as median (IQR). Figure S2: Correlations between EPP-QoL and PROMIS-57 domains (A–D), PPIX and time to symptoms (E,F), and time to symptom onset and QoL scores (G–J). (A–C) EPP-Qol total score correlated with the PROMIS-57 Social Function score, Physical Function score, and Depression score. (D) EPP-QoL outdoor activity limit correlated with the PROMIS-57 Social Function score. (E,F) Metal-free erythrocyte protoporphyrin level did not correlate with time to symptom onset before or during afamelanotide treatment in patients with protoporphyria. (G) Time to symptom onset during treatment correlated with EPP-QoL score. (H) Time to symptom onset during treatment correlated with PROMIS-57 Social Function score. (I) Time to symptom onset during treatment correlated with PROMIS-57 Depression score. (J) Time to symptom onset during treatment did not correlate with time to symptom onset before treatment. Time to symptom onset and metal-free erythrocyte protoporphyrin levels were natural log-transformed before measuring associations. Figure S3: There was no change in aspartate aminotransferase (AST) with afamelanotide treatment.
