# Supplementary material for: Afamelanotide for Treatment of the Protoporphyrias: Impact on Quality of Life and Laboratory Parameters in a US Cohort
Source: Life (Basel). 2024 May 28;14(6):689. doi: 10.3390/life14060689 (PMC11204624; doi:10.3390/life14060689)

Supplementary material

Supplemental Figure S1: Changes in individual EPP-QoL and PROMIS-57 questions were sustained over time during treatment with afamelanotide. (A) Improvement in well-being was sustained over time. (B) Improvement in outdoor activities limitation was sustained over time. (C) Improvement in QoL was sustained over time. (D) there was a non-significant trend toward improvement in EPP skin complaints over time. (E) Improvement in Social Function was sustained over time. (F) Improvement in Physical Function was sustained over time. (G) Decrease in depression was sustained over time. (H) Pain increased over time. Data for all panels are shown as median (IQR).

Abbreviations: EPP-QoL, Erythropoietic protoporphyria quality of life tool; IQR, interquartile range (25th-75th percentile). PROMIS-57, Patient Reported Outcomes Measurement Information System 57; QoL, quality of life.

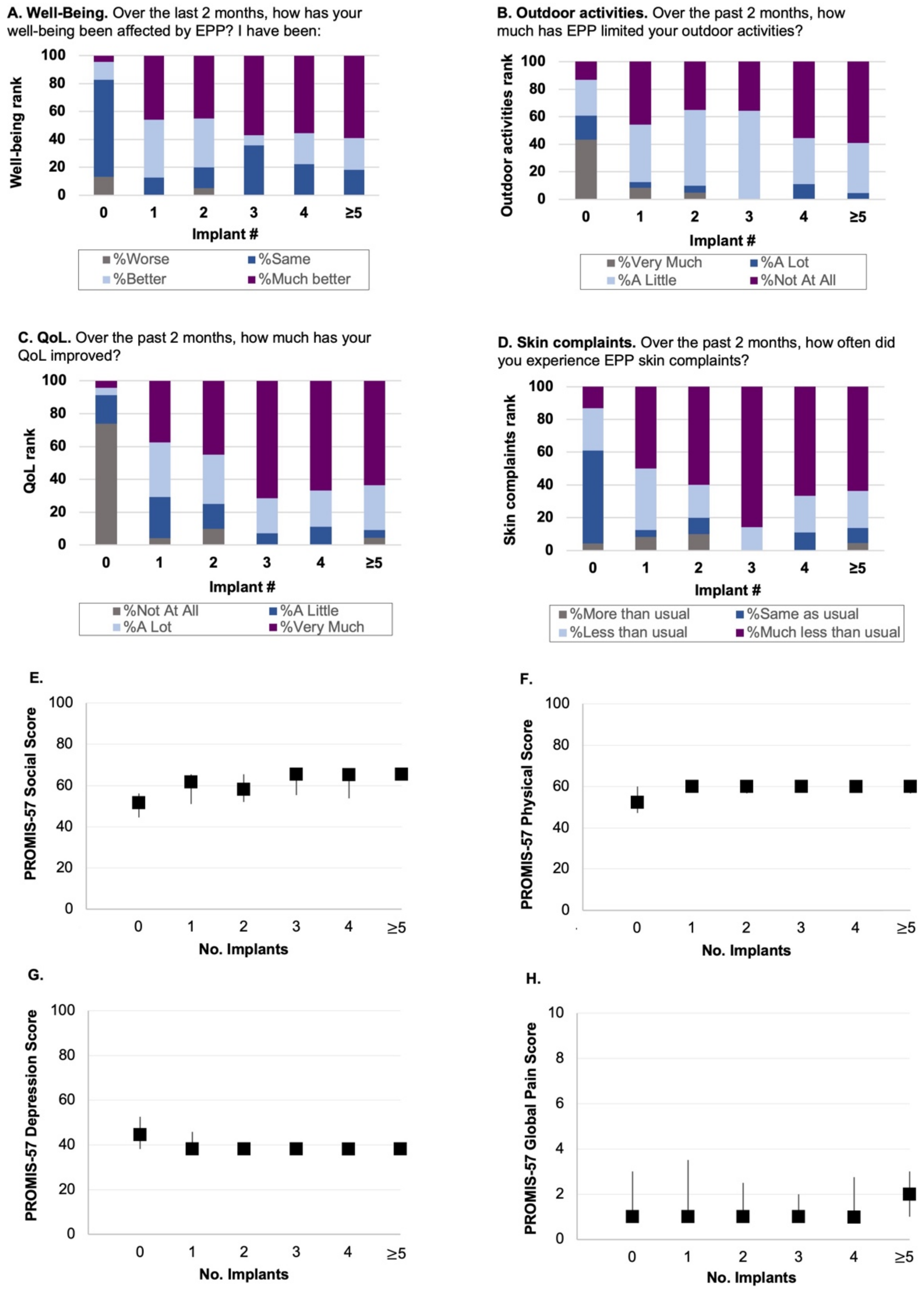

Supplemental Figure S2: Correlations between EPP-QoL and PROMIS-57 domains (A-D), PPIX and time to symptoms (E,F), and time to symptom onset and QoL scores (G-J). (A-C) EPP-Qol total score correlated with the PROMIS-57 Social Function score, Physical Function score, and Depression score. (D) EPP-QoL outdoor activity limit correlated with the PROMIS-57 Social Function score. (E,F) Metal-free erythrocyte protoporphyrin level did not correlate with time to symptom onset before or during afamelanotide treatment in patients with protoporphyria. (G) Time to symptom onset during treatment correlated with EPP-QoL score. (H) Time to symptom onset during treatment correlated with PROMIS-57 Social Function score. (I) Time to symptom onset during treatment correlated with PROMIS-57 Depression score. (J) Time to symptom onset during treatment did not correlate with time to symptom onset before treatment. Time to symptom onset and metal-free erythrocyte protoporphyrin levels were natural log-transformed before measuring associations. **Abbreviations:** EPP-QoL, Erythropoietic protoporphyria quality of life tool; PROMIS-57, Patient Reported Outcomes Measurement Information System 57; PPIX, protoporphyrin IX.

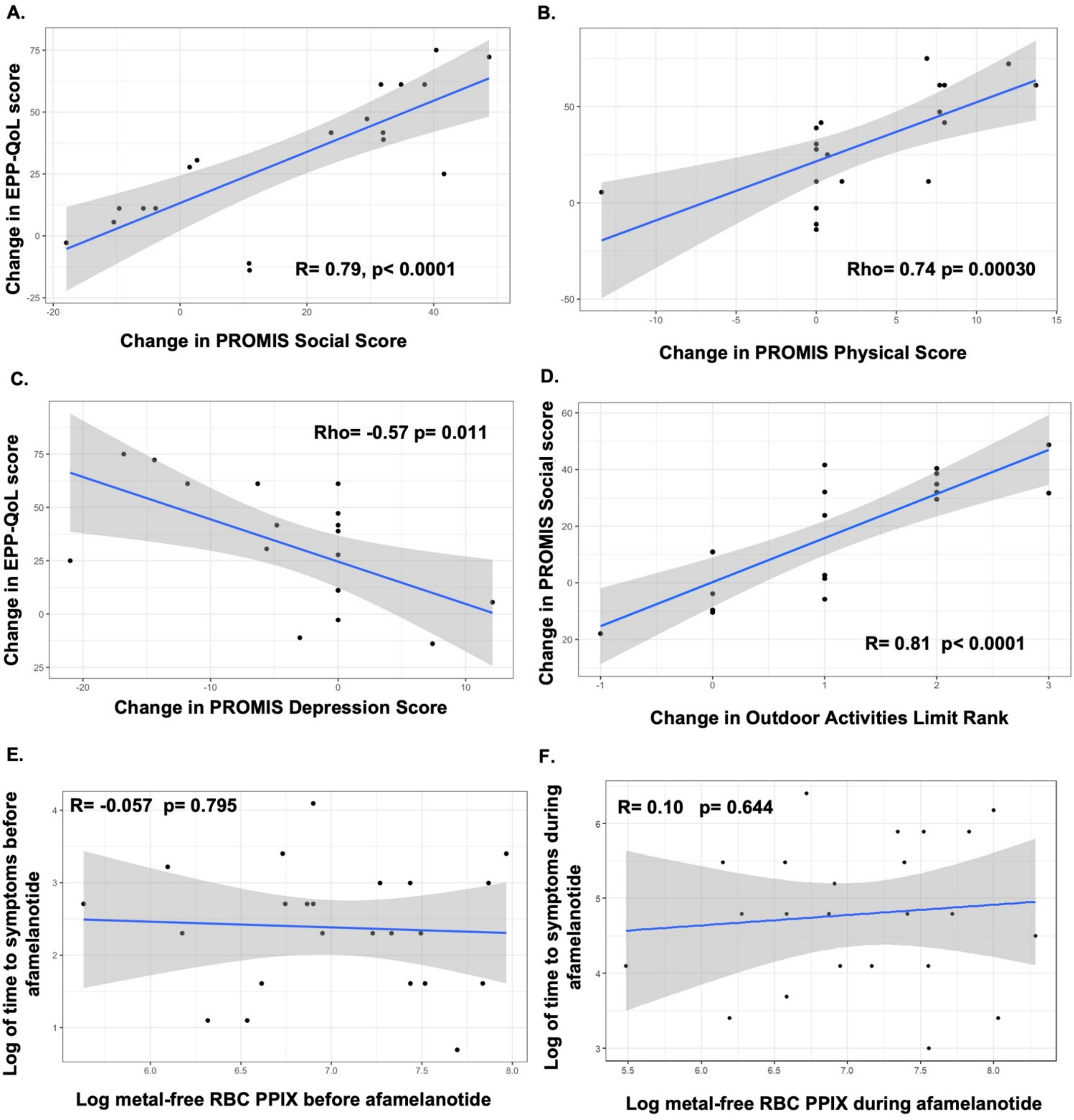

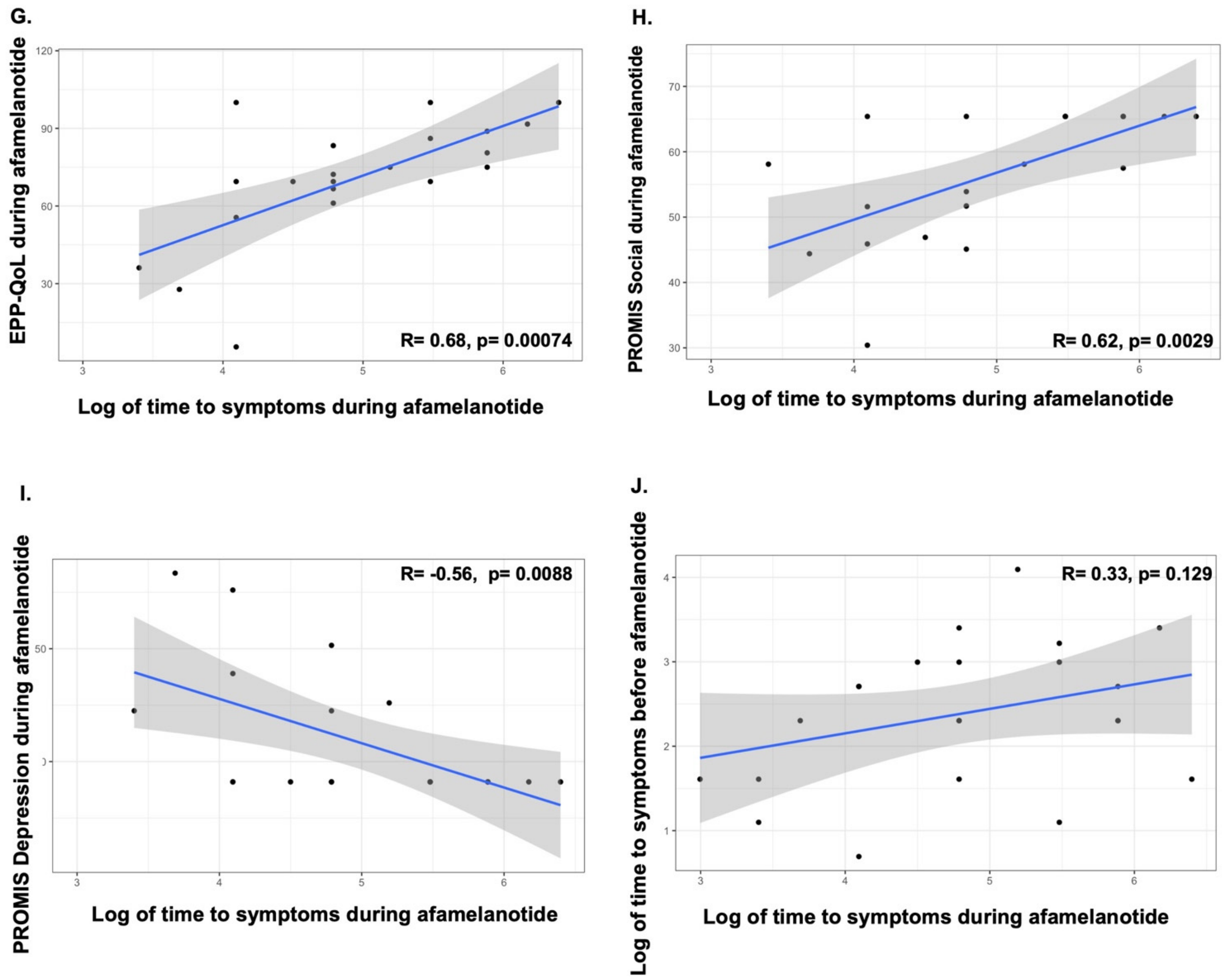

Supplemental Figure S3: There was no change in aspartate aminotransferase (AST) with afamelanotide treatment.

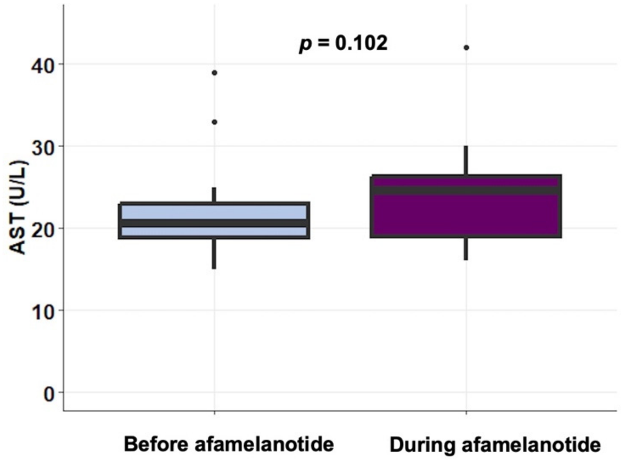

Supplement: Supplementary file 1 [file life-14-00689-s001.zip › life-3009828-supplementary.pdf]
